# Practical Notes on Diagnosis and Treatment

**Published:** 1907-08-03

**Authors:** 


### Practical Notes on Diagnosis and Treatment.

### Chronic Alcoholism.

Chlorine water has been credited with the power both to cure alcoholic gastritis and to check the craving for drink. It may be prescribed as follows:—Aq. chlori 8 parts, decoct. althææ 165 parts, sacch. alb. 8 prats. A dessertspoonful every two or three hours.

### Lead Lotions in Eye Injuries.

Whether you know the cornea is damaged or not, always avoid all applications containing lead. A slight injury may be easily overlooked, and if lead is applied to a wounded cornea an opaque white deposit is formed on the abraded surface, and should this happen to be centrally situated it materially interferes with vision.—*Mr. J. B. Lawford.*

### Keloid.

This is an overgrowth of connective tissue in a cicatrix. It may be limited to the scar or may extend into healthy tissue. Sometimes it forms in connection with vaccination marks. It is a sort of hypertrophy of cicatricial tissue. The most important point in connection with treatment is that in no circumstances should the keloid be excised. If this is done the condition will return in an aggravated form. The best treatment is electrolysis.

### Early Operation.

If you would save your patient who is suffering from acute intestinal obstruction, you must operate early. It is quite possible that if you carry out this rule you may some day perchance operate on a patient unnecessarily. Well, if you do, it will be a matter of profound regret. But I would sooner open a patient's abdomen unnecessarily than I would delay an operation too long and find that when I did operate I was too late.—*Mr. Pickering Pick.*

### Local Application in Gout.

To relieve the pain warm packs should be arranged round the affected joint. These should consist of cotton wool saturated with a soothing lotion and then lightly covered with oil silk. The following is a useful formula: Sodium carbonate 3 drams, belladonna liniment 2 ozs., tincture of opium 2 ozs., water to 8 ozs. Mix a small quantity of this with an equal quantity of hot water, and then pour on the cotton wool, previously arranged round the joint. The pack should be changed every four hours.—*Dr. Luff.*

### Pain in Spinal Caries.

In connection with caries of the spine, remember that persistent pain around one side of the trunk at a definite level is seldom due to any other cause than organic irritation of the nerve roots. Neuralgic pains of the trunk are fugitive, inconstant, varying in time and often also in place; the pains of organic disease are persistent in the same spot. Another point to remember is the necessity in any doubtful case for frequent examination of the spine for conclusive evidences of bone disease. A negative result on one examination is not sufficient; the examination should be repeated at least once a fortnight.—*Sir Wm. Gowers.*

### Graves's Disease.

Faradism to the pneumogastrics—one pole on the neck, the other over the heart—has in some cases caused considerable improvement in exophthalmic goitre.

### Catheterism.

Always administer a dose of quinine, with morphine or potassium bromide, after passing an instrument, and place the patient in a warm bed if possible. In this way high temperatures may be avoided.—*Mr. A. G. Miller.*

### Laxative for Infants.

Sulphate of magnesium dissolved in simple syrup is a useful laxative for infants. Five to ten grains may be ordered once, twice, or thrice daily. Greater activity may be ensured by replacing part of the simple syrup by syrup of senna.

### Multiple Warts.

It is said that multiple warts will often disappear under the internal administration of lime-water. A wineglassful may be taken three times a day, or even oftener, and the patient must be encouraged to persevere with the treatment for several weeks.

### Erysipelas of the Face.

As a local application a saturated solution of camphor in ether has been recommended. It is said rapidly to relieve the pain and inflammation. The inflammable nature of the solution calls for obvious precautions.

### An Artificial Sulphur Water.

As a substitute for one of the natural sulphur waters the following may be tried: Dissolve ʒiiss. of crystallised hyposulphite of sodium in ℥x. hot distilled water and add ℥v. glycerine. A teaspoonful mixed with a quart of water charged with carbonic acid gas forms instantly an artificial sulphur water.

### Snuff for Coryza.

The following is a useful formula: Salol 1 part, salicylic acid 20 parts, tannic acid 10 parts, boric acid 4 parts. A pinch should be introduced into each nostril on the commencement of the symptoms, and repeated every hour for eight hours, but not longer.

### Tubercular Glands.

These in the cervical region are often enclosed in a well-marked thickened sheath, or capsule, and if the surgeon does not very carefully define this sheath and lay it open fully, he will fail to enucleate the gland quickly. By keeping just inside the sheath he will be able to shell out the glands easily, and without risk of bleeding.—*Mr. Stephen Paget.*

### The Use of Mydriatics.

In patients over 40 years of age mydratics carry some risk of setting up glaucoma. Hence before their use it is well to ascertain the tension of the globe; to inquire as to the presence of prodromata of glaucoma, such as rainbow colours, etc.; to employ weak solutions when practicable; and to instil afterwards either eserin or pilocarpin (myotics).—*Mr. Simeon Snell.*

